# Supplementary material for: Neglected tropical disease meets neglected community: Street children’s susceptibility to scabies in Addis Ababa, Ethiopia
Source: PLoS Negl Trop Dis. 2024 Sep 6;18(9):e0012475. doi: 10.1371/journal.pntd.0012475 (PMC11410222; doi:10.1371/journal.pntd.0012475)
Supplement: Attachment — Submitted filename: Comments_Reviewer 4.docx [file pntd.0012475.s001.docx]

Comments

1. *A range of health complications associated with scabies have been reported, including pain, itching, systemic complications from secondary bacterial infections, ~~nephritis~~, rheumatic fever, septicemia, glomerulonephritis, and rheumatic heart disease (Hay et al., 2012; Kaburi et al., 2019; Welch et al., 2021), ~~abscesses~~ and kidney disease (Azene et al., 2020).*

Itching is a symptom of scabies and not complication.

Pain may be present in the presence of intense scratching or secondary pyoderma.

Nephritis and abscesses have been mentioned twice

2. *and has a 5–10% fatality rate per year*

Crusted scabies could be mentioned

3. *Street girls constituted less than a quarter of the total participants.*

In the results paragraph, please start with the total number of participants. and then the percentage of girls. Details pertaining to the number and percentage of boys , age distribution, youngest and oldest age, education status of boys vs girls, duration of infection before initiation of treatment, crusted scabies, any complication need to be mentioned.

4. Manuscript could be revised and made concise, with references being inserted at the end of the sentences and not mentioned within the brackets
5. References to be mentioned in Vancouver style
