# Supplementary material for: Neglected tropical disease meets neglected community: Street children’s susceptibility to scabies in Addis Ababa, Ethiopia
Source: PLoS Negl Trop Dis. 2024 Sep 6;18(9):e0012475. doi: 10.1371/journal.pntd.0012475 (PMC11410222; doi:10.1371/journal.pntd.0012475)
Supplement: Attachment — Submitted filename: Response letter_Revised final for submission.docx [file pntd.0012475.s002.docx]

**Dear editor,**

We want to take a moment to express our sincere gratitude for the editorial team’s support and guidance throughout the review process of our manuscript. Additionally, the feedback and suggestions we received from the reviewers were truly invaluable, and we are grateful for the time and effort they invested in helping us to improve our work. We have read through all the comments and made the necessary revisions to the manuscript.

Here are the major issues addressed in response to reviewers’ comments:

- The manuscript has been formatted according to the authors’ guideline, the most notable of this being changing both the in-text citations as well as the list of references to the Vancouver style. We have also included some of the materials that were cited in the text but missing in the reference list.
- The methodssection, especially selection of study participants and method of data collection and analysis has been substantially improved.
- Issues related to grammatical errors in some parts of the manuscript have been addressed and the entire manuscript has been thoroughly edited by a native English speaker in UK.
- Further to a suggestion from the editors (after quality check), we have included authors’ summary
- All the concerns in the introduction, results, discussion, and conclusion sections areaddressed below.

We have provided point-by-point responses to the comments of the reviewersbelow. Further to the request of the editorial team, we have submitted both the revised version with yellow color highlighting the changed areas according to the comments and another clean version.

Should you have any questions or need further clarification, please do not hesitate to contact me (the corresponding author) at any time.

| Reviewer | Comments/questions | Authors’ response |
| --- | --- | --- |
| 1. | **Methods:**  **Selection of study participants:**   1. The authors talk of collecting data about the embodied experience of scabies infestation, street children who had a history of a previous scabies infestation or those who were infested with it during the interview. Was scabies diagnosed clinically (clinician) or just the mere presence of scabies-like skin manifestation? The reader needs to be sure that these street children actually had scabies and not the differential diagnosis (other skin infestations) of scabies. | - We did not conduct clinical diagnosis of scabies, but were interested in what the participants considered to be scabies. However, for those who disclosed that they had a history of infestation but had recovered, we checked with the healthcare provider to confirm that their condition had been scabies. We were able to do this because most participants were treated in modern health facilities. Some participants with scabies said that they had experiencedtwo or more rounds of infestation after having received treatment. Others were receiving treatment after being clinically diagnosed by a trained professionalat the time of data collection. |
|  | 2. It will be useful for the authors to list the inclusion/exclusion criteria first before explaining each of them. | - The major inclusion/exclusion criteria were 1) being a child of the street, 2) being below 18 years old at the time of data collection, and 3) reported history of scabies infestation. Thank you for your suggestion, this has now been included in the revised version (P4, lines 115-117). |
|  | **Method of data collection and analysis:**   1. The statement “Qualitative data were collected using an in-depth interview method” should read “Qualitative data was collected using in-depth interviews” | - We prefer to stick with using ‘data’ as a plural noun, hence “Qualitative data were collected using in-depth interviews”. The singular form ‘datum’. |
|  | 2. It will be important to indicate what is contained in the interview guide or refer the reader to it as a supplementary file. | - We haveindicated what is contained in the interview guide (P5, lines 138-156). If need be, we can also provide the interview guide as a supplementary file. |
|  | 3. Was the interview guide written in the local language or in another language and translated? If you so was the translation done by a professional or how was it done? | - The interview guide was first written in English. It was then translated into the two local languages (Amharic and Afaan Oromoo) predominantly spoken in the area. The translation was done by the first two authorswho are fluent in these languages. We have also made back translation. |
|  | 4. Were the interviews conducted in the entire December 2023? The authors need to be specific with the dates. | - Data collection was undertaken from 3-28 December, 2024 and this has been specified in the revised manuscript (P5, line 166). |
|  | 5. The statement “The number of children interviewed was determined based on the principle of theoretical data saturation” is not clear. The need to explain what this theory means in the context of this article and indicate the source as well. | - Data was considered saturated when no new elements were found and the addition of new information was no longer necessary, since it would not alter the comprehension of the researched phenomenon (Nascimento et al., 2018). This has been added to the revised version, thank you for your suggestion (P6, lines 184-186). |
|  | 6. Why were most participants getting bored after some minutes of conversation? | - As indicated in the article, most of the street children in the study area were highly addicted, especially to glue. Because the interviewer understood that they becamesleepy while sniffing the glue, he had to ask them not to use the glue during the interview. In addition, given the children’s highly mobile lifestyle, they rarely sit for 20-30 minutes in one place. Although the interviewer tried to make the interview process informal, it was a common problem among most participants to get bored after some minutes of the interview. |
|  | 7. The data analysis section needs to be rewritten well. For instance how did the authors manage issues of disagreement during coding? It is also not clear how the main themes and sub-themes were arrived at. | - In a bid to reduce the challenge of subjectivity and provide a sound interpretation of data, the data analysis involved collaborative coding. Although much of the iterative process of coding and interpretation of the data ended with an acceptable agreement among the coders, some of the inter-coder disagreements were resolved through discussion. This has been included in the revised version (P6, lines 195-198). |
|  | 8. Was data analysis done inductively or deductively? This needs to come out clearly. | - We employed a mixture of inductive and deductive approaches to coding. The data collector started setting codes deductively based on the interview guide and field notes. This was followed by inductively developing new codes and iterating on the existing ones as the transcripts were sifted through (P6, lines 192-195). |
|  | **Results:**   1. It will be important to include some percentages in the socio-demographic characteristics. | - We have included some percentages in the socio-demographic section (P7, lines 226-232). Thank you for the suggestion. |
|  | 2. It will also be useful for the authors to organize the results into main and sub-themes and clearly stated as such. | - Corrected accordingly (P7-16 lines 225-566), thank you! |
|  | 3. This section is poorly organized. | - We have tried to amend the manuscript according to your suggestions and those of other reviewers. |
|  | 4. It is better to present some of the results as figures or percentages so that the reader gets to know what proportion of the participants expressed their views of a particular finding | - While we have tried to include some percentages in the socio-demographic characteristics of the participants, the qualitative nature of the study made us emphasize nuances rather than quantification of responses. |
|  | **Susceptibility to Scabies**   1. Please check “•••”(‘ekek’). | - Checked, thank you! |
|  | 2. What makes it clear that street children in Addis Ababa are susceptible to scabies? | - Changed to “The study indicated that street children in Addis Ababa are susceptible to scabies.” –P7 lines 240 |
|  | 3. The authors use the term prevalence loosely. How is susceptibility related to prevalence? The authors must realize that prevalence is a technical term and should be treated as such. | - Understanding that the term ‘prevalence’ is better confined to quantitative studies, we have replaced it with “scabies appeared to be common among street children” in others –P8, lines 259-266 |
|  | 4. Not too sure how physical, social and psychological impacts fall under susceptibility. Similarly same applies to social consequences with psychological consequences. | - Susceptibility is a broad concept that includes not only participants’ exposure to the disease but also their vulnerability to bear the burden of the condition, such as psycho-social impacts. We have considered susceptibility to apply to all these areas, rather than only to the epidemiology of the condition or health seeking behavior. |
|  | 5. Why talk about prevalence here when the objective of the paper was to explore the susceptibility to scabies and related health-seeking behavior of street children in Addis Ababa?   Did the headings: The role of mutual support or social capital as a street sub-culture; The role of free healthcare services; and Anticipated and experienced negative social reactions from significant others emerge from the data or were predetermined? | - Yes, the headings: The role of mutual support or social capital as a street sub-culture; The role of free healthcare services; and Anticipated and experienced negative social reactions from significant others emerged from the data, they were not predetermined. |
|  | **Discussion**   1. The statement “This study showed that street children in Addis Ababa and possibly in the entire country are highly susceptible to scabies” cannot be arrived at from this type of study. | - We agree with you that it is difficult to arrive at this conclusion from this study. Accordingly, this has been corrected as “This study showed that street children in Addis Ababa are highly susceptible to scabies.” (P16, line 568 |
|  | 2. The statement “They reported frequent infestations, which was borne out by observation” is not clear. | - For clarity, we have changed the statement into: “Street children reported frequent infestations” and this has been included in the revised version. (P16, line 570) |
|  | 3. Since this is not a prevalence study it is inappropriate to compare prevalence of scabies in other areas to that of this study. | - As this is not a quantitative research, we have replaced the term “prevalence” or “incidence” by other phrases such as ‘scabies is common among the study population’. (P16, line 564-565) |
|  | **Conclusion**:   1. The study design and limitations of this study does not allow for the statement that “This study has uncovered considerable morbidity ascribable to a neglected tropical disease among a neglected social group” | - We have corrected this as: “This study suggests that scabies is common in this neglected social group.” P 19, Line 659 |
|  | 2. The conclusion should be a brief summary of key findings. | - A brief summary of the findings and whether they were consistent with the findings of other studies has been presented in the Discussion section. Consequently, we prefer to conclude what has been generally learnt from the study in relation to its objective. Thanks for understanding! |
|  | 3. The recommendation is not specific and non-targeted. Whom are the recommendations meant for? | - We have added the following into the Conclusion section: “In this regard, both governmental and non-governmental organizations working on street children should strengthen interventions focusing on sustainably reunifying and reintegrating street children.” (P19, lines 662-665) |
|  | **Summary and General Comments:**   1. The manuscript is saddled with lots grammatical errors and will need substantial English editing. | - The manuscript has been edited by a native English speaker who is also among the authors of the manuscript. But, we understand that this does not guarantee perfection and we will consider revision, including during the proof reading. |
|  | 2. The referencing style of the manuscript doesn’t appear to be that recommended by Plos journals. | - Thanks for this comment - both the in-text citations and list of references have been changed into the “Vancouver” style, according to the authors’ guideline of the journal. |
|  | 3. Line numbering is recommended would have aided easy review. | - Noted and added |
|  | 4. What is the quote (just before background) of page 2 meant for? | - We have moved this quote to the Results section (understandings about the epidemiology of scabies) and explained it there. (P10, lines 343-345) |
|  | 5. The entire paper needs extensive formatting. | - We have made utmost effort to format the paper according to yours and other reviewers’ suggestions and the journal’s guidelines. |
|  | **Background:**   1. It will be useful to indicate the current prevalence of scabies in Ethiopia instead of stating scabies as being the most common diagnosis in the statement “weighted prevalence of clinically confirmed skin diseases in Ethiopia is 22.5%, with scabies being the most common diagnosis” | - We have made corrections according to your suggestion. (P3, lines 64-66) |
|  | 2. In the last paragraph the authors state that “The few existing studies are dominated by medical perspectives, and studies undertaken from social science perspectives are lacking”. This justification is not clear or convincing enough. Social science perspective is too broad and hence the need for the authors to be very clear on this. | - Social science research around NTDs is essential if key biomedical advances are to be successfully translated into elimination and control programmes. Most of the studies undertaken on scabies in Ethiopia, are dominated by issues related to prevalence rate and associated factors. These studies, though important or useful, do not focus on scabies in relation to the socio-economic position of the studied population and their lived experiences. Therefore, it is justifiable to argue that social science perspectives, including those from sociology and anthropology, are needed to understand the situation and improve interventions. |
| 3. | **Results**:  In the results paragraph, details pertaining to the total number, number and percentage of boys vs girls, youngest age, oldest age have to be mentioned. results have been presented in a narrative pattern | - We have added percentages regarding the socio-demographic characteristics of participants to the results section (P7, lines 226-232) |
|  | **Conclusion:**  The authors have highlighted the immediate need to address this NTD infection with due importance given to rejoining the street children to their family/homes and deaddiction from glue. There is an urgent need to find out if the street children in the other states have access to treatment care as per National policy guidelines. The results indirectly point out to the need to address water scarcity. | - The feedback is well noted. The suggestion to undertake further study to uncover whether street children in other areas of the country have access to healthcare service as per the national policy guidelines is well taken. Currently, the same team is undertaking a study at national level to understand these and other issues. |
|  | **Summary and General Comments:**  Please address the comments mentioned alongside the manuscript. The manuscript could be made more concise. References need to be inserted, rather than mentioning the authors within the brackets References could be mentioned in  Vancouver style | - We have changed both the in-text citations and the references to Vancouver style as you suggested and according to the journal’s guideline. |
