# Supplementary material for: Neglected tropical disease meets neglected community: Street children’s susceptibility to scabies in Addis Ababa, Ethiopia
Source: PLoS Negl Trop Dis. 2024 Sep 6;18(9):e0012475. doi: 10.1371/journal.pntd.0012475 (PMC11410222; doi:10.1371/journal.pntd.0012475)
Supplement: Attachment — Submitted filename: PNTD-D-24-00471_Review2.docx [file pntd.0012475.s003.docx]

**Reviewer comments**

I am generally satisfied with the substantial efforts made by the authors to improve this manuscript. I however suggest the following minor corrections:

Methods and materials

Selection of study participants

1. Based on the response by the authors concerning the diagnosis of scabies, I suggest they reframe the sentence “those who considered themselves to be infested with it during the interview” in lines 120-121 to “those infested with it during the interview”
2. I am still a bit confused about the statement “In addition, street children with no history of infestation were also included” in line 122. I thought the authors are concerned about lived experiences of street children who previously and at the time of interview were infested with scabies. If so then that statement should be left out and 3) in line 118 rephrased as “reported history of or current scabies infestation”

Method of data collection and analysis

1. I suggest the authors’ incorporate the responses to the interview guide into the main manuscript.
