# Supplementary material for: Neglected tropical disease meets neglected community: Street children’s susceptibility to scabies in Addis Ababa, Ethiopia
Source: PLoS Negl Trop Dis. 2024 Sep 6;18(9):e0012475. doi: 10.1371/journal.pntd.0012475 (PMC11410222; doi:10.1371/journal.pntd.0012475)
Supplement: Attachment — Submitted filename: response letter 2.docx [file pntd.0012475.s004.docx]

**Dear editor,**

Once again, we want to take a moment to express our sincere gratitude for the editorial team’s support and guidance throughout the review process of our manuscript. Additionally, the feedback and suggestions we received from the reviewers were truly invaluable, and we are grateful for the time and effort they invested in helping us to improve our work. We have read through all the comments and made the necessary revisions to the manuscript.

Here are the major issues addressed in response to reviewers’ comments:

1. Based on the suggestion of the reviewer, we have reframed the sentence “those who considered themselves to be infested with it during the interview” in lines 120-121 to “those infested with it during the interview”
2. As you know, the study mainly aimed to understand the perceived susceptibility to scabies and related health seeking behavior of children of the street in Addis Ababa. Attempt to understand the perceived susceptibility to scabies and related responses (primary health seeking behavior-preventive activities) requires perspectives both from those with history of infestation and the children that have not been infested with scabies. Obviously, lived experiences of infestation, including stigma, and responses to infestation- [secondary] health seeking behavior requires collecting data from the children with active or previous history of infestation. Accordingly, we admit that considering history of infestation as one of the inclusion criteria was wrong and hence, we have omitted this from the manuscript.
3. Dear reviewer, the suggestion to “incorporate the responses to the interview guide into the main manuscript” is not clear. Perhaps, if our understanding that you suggest us to incorporate the transcripts (raw data) to the main document is correct, we have already included our data sharing statement as requested by the system. Similarly, if you want us to share our interview guide (tool) as supplementary file, that is also okay and we can do that in consultation with the editorial team.

Looking forward to your swift final decision as always!

Best,

Bewunetu (on behalf of the team)
